# Novel dual-function CellDetect^® ^staining technology: wedding morphology and tinctorial discrimination to detect cervical neoplasia

**DOI:** 10.1186/1746-1596-5-70

**Published:** 2010-11-11

**Authors:** Pavel Idelevich, Adi Elkeles, Elimelech Okon, Don Kristt, Dov Terkieltaub, Ilia Rivkin, Ilan Bruchim, Ami Fishman

**Affiliations:** 1 Zetiq Technologies Ltd., Ramat Gan, Israel; 2 LEM Pathology Laboratory, Nes Ziona, Israel; 3 Molecular Pathology Unit, Rabin Medical Center, Petah Tikva, Israel; 4 Department of Obstetrics and Gynecology, Meir Medical Center, Kfar Saba, Israel, affiliated with Sackler School of Medicine, Tel-Aviv University

## Abstract

**Background:**

A persistent goal of oncologic histochemistry is to microscopically identify neoplasia tinctorially. Consequently, the newly developed CellDetect^® ^staining technology, that appears to exhibit this property, warrants clinical evaluation. The objective of this study was to compare the diagnostic results using CellDetect^® ^to the outcomes of standard microscopic examination based on hematoxylin and eosin (H&E) staining for the recognition of different squamous epithelial phenotypes of the uterine cervix.

**Methods:**

Pairs of adjacent sections were made from 60 cervical biopsy cases that were diagnosed originally as either normal or neoplastic (CIN, SCC). One section of the pair was stained for H&E; the second section, with CellDetect^®^. Based on the examination of these pairs by two experienced pathologists, we investigated the following issues:(1) diagnostic agreement between the pathologists on each pair; (2) agreement between H&E and CellDetect^® ^for each pair (3) tinctorial characteristics in micro-regions (n = 130) evaluated as either normal, reactive or neoplastic.

**Results:**

Qualitatively, CellDetect^®^-stained preparations displayed cyto-morphological detail comparable to H&E images. Tinctorially, *non-neoplastic *cells appeared green/blue when stained withCellDetect^®^, contrasting with cytologically *neoplastic *foci, where cells of every grade were red/magenta in color. Due to these tinctorial characteristics, even small foci of neoplasia could be readily distinguished that were inconspicuous on H&E at low magnification. In some instances, this prompted re-examination of the H&E and revision of the diagnosis. Quantitatively, we found that despite diagnostic variation between pathologists, in about 3% of the cases, each pathologist made the same diagnosis regardless of whether CellDetect^® ^or H&E was used, i.e. there was 100% self-agreement for each pathologist between stains. Particularly noteworthy was the finding of a 0% false negative rate, coupled with a 10-15% false positive rate. Regarding specificity, the performance in *reactive *squamous processes was similar to that observed for morphologically normal squamous epithelium.

**Conclusions:**

In this first order assessment of clinical applicability, CellDetect^® ^staining technology was at least comparable to results using H&E, and perhaps surperior. CellDetect^® ^provided a uniquely useful tinctorial clue for the detection of neoplasia, which exhibited an impressive 0% false negative rate. A more extensive, blinded study is needed to confirm these promising findings.

## Background

Although cervical cancer is still one of the major sources of mortality in women, advances have been made in combating this disease by early detection and diagnosis [1]. Cytological screening programs that triage suspicious epithelial changes for subsequent definitive biopsy have had a significant impact on achieving improved therapeutic outcomes. Nonetheless, an assessment of cell or tissue morphology alone may be inadequate to detect early indications of neoplasia in some cases. Due to this limitation of a solely histological approach, a range of histochemical, immunocytochemical, and molecular biological approaches have been used as adjuncts to standard microscopy [2,3]. The latter methods add information on the unique cell biology believed to distinguish a neoplastic phenotype [2,4]. However, these adjuncts are not generally informative on all, or even most, neoplastic conditions. We thus remain challenged to find a universal light microscopic diagnostic tool for neoplasia, particularly for recognition of *in situ*, pre-malignant states that represent the most urgent goal of early detection programs.

Recently, Zetiq Technologies Ltd. introduced a new morpho-histochemical stain for neoplasia, named CellDetect^®^. This staining technology has been shown to consistently differentiate cancer from normal tissues and reactive states in histological and cytological preparations, as well as cultured cell lines [5,6]. In such stained preparations, the cytoplasm of normal cells is green-blue, whereas the cytoplasm of neoplastic elements is distinctively pink-red or magenta. These differences can be objectively demonstrated using optical instruments that discriminate wavelength [5]. Importantly, the morphological characteristics of cells stained with CellDetect^® ^are preserved, allowing the application of standard, microscopic diagnostic criteria as well. This background suggested that CellDetect^® ^might serve as a useful adjunct in clinical applications, since it offers the morphological resolution capabilities of hematoxylin and eosin (H&E) staining, while providing an additional histochemical dimension for the diagnosis of neoplasia.

Consequently, we undertook this first-order, non-blinded evaluation of CellDetect^®^'s performance in assessing cervical epithelial neoplasia. Towards this end, the CellDetect^® ^stain was applied to a series of clinical samples representing different phenotypic states of the cervical squamous epithelium, from normal to invasive SCC. The diagnosis made on each CellDetect^®^-stained section was compared to the results on an adjacent section stained with standard H&E. In order to avoid confounding errors due to intra-observer variability, each pair of slides from all cases was independently assessed by two pathologists. We found that the diagnostic results using CellDetect^® ^were completely comparable to H&E. In one regard CellDetect^® ^staining was superior because the tinctorial characteristics preferentially facilitated judging the grade of intra-epithelial neoplasia microscopically, even at low magnification.

## Methods

This was a single-center, non-blinded study using 60 archival cervical biopsy cases with the following diagnoses: normal, 22 cases; CIN-2/3, 18 cases; squamous cell carcinoma, 20 cases. To assess the staining in morphologically *reactive processes, 12 *micro-regions (see below), in these cases, were specifically examined.

All cases were diagnostically re-evaluated using two new adjacent sections that were cut from a single paraffin block representative of each case. One of these two sections was stained by the standard H&E procedure, and the other by the CellDetect^® ^method. All of these pairs of stained slides were reviewed by two pathologists, independently; their diagnostic conclusions for each case were compared in regard to: (1) the two stains as a function of diagnosis and (2) inter-observer agreement for diagnosis in each case.

### Staining Protocols

#### Hematoxylin/Eosin Staining Protocol

Paraffin-embedded tissue samples were sectioned at 4 microns, and mounted on standard microscopic slides. Deparaffinization was accomplished as follows: immersions in xylene, twice (3 min each); 100% ethanol, twice (3 min each); immersion in 95% ethanol, twice (3 min each); distilled water (3 min). These sections were stained with hematoxylin, Dye-1, (1 min), washed in tap water until the water was clear. Sections were stained in eosin, Dye-C, (1-2 min) and washed in tap water, as above. For cover-slipping, the sections were dehydrated in an ascending series of ethanol concentrations (50%, 70%, 80%, 95% × 2, 100% × 2), cleared in xylene (3 - 4 times), and then mounted in Permount, or an equivalent commercial product.

#### CellDetect^® ^Staining Protocol

The staining was performed according to the manufacturer's instructions. Although the details of the protocol and the formulation of the stains are currently proprietary information, the kits contain three ingredients: (1) an acidophilic red stain, (2) a basophilic green stain, and (3) a proprietary plant extract necessary for the specific reaction. The six-step staining protocol was completed in approximately 30 min.

### Slide Evaluation

This was made in two stages.

*(1) Comparison of observer performance: *Each of the two experienced pathologists examined each case with both stains, and made two observations. First, an overall diagnosis on a case was made. Second, they recorded whether their own diagnostic impression on the H&E was in agreement with their impression based on the CellDetect^®^-stained slide.

*(2) Comparison of individual micro-regions: *Morphological versus tinctorial performance of *CellDetect*^® ^relative to H&E, was assessed in multiple, small regions within each of the 60 biopsies that exhibited a uniform histological phenotype. These are referred to as: *micro-regions*. Since the regional variation along the squamous epithelium in each case ranged from normal, CIN or SCC, multiple morphologically distinct regions were present in most cases. The total number of regions was 130. For each region, a single pathologist first assessed the morphology using H&E. Then independently, the same region was examined in the adjacent section stained with CellDetect^®^. Thus two morphological diagnoses were made on each region, which were compared to the tinctorial characteristics in CellDetect^® ^of each site.

## Results

### Qualitative features of CellDetect^® ^staining

**Figure 1:**
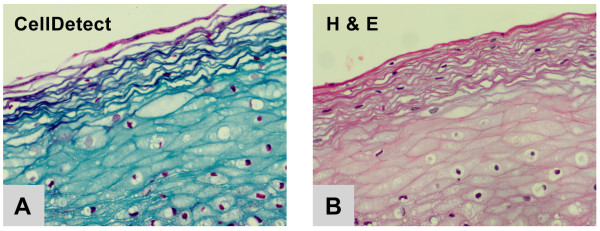
**CellDetect**^® ^**and H&E staining of normal cervical epithelium**. Using CellDetect^® ^normal epithelial cells stain green/blue.

**Figure 2:**
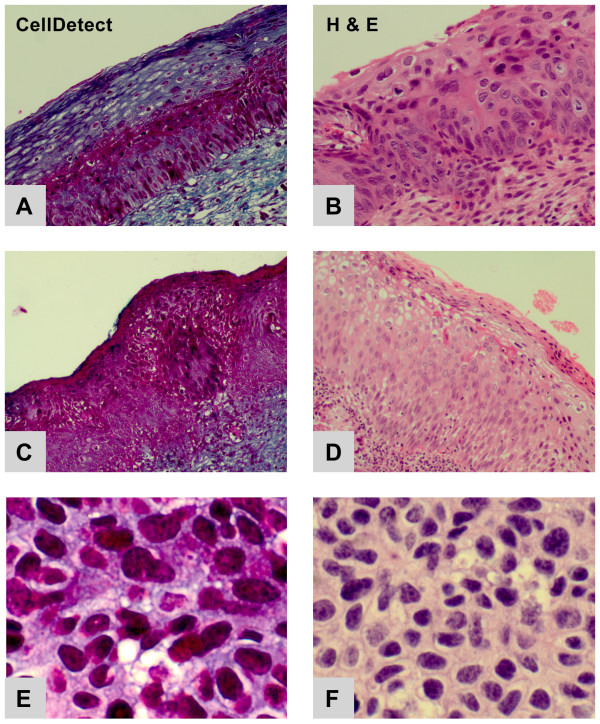
**CellDetect**^® ^**(A, C, E)and H&E (B, D, F) staining of neoplastic cervical epithelium**. CellDetect^® ^stained neoplastic cells red/magenta, at every grade. (A) CellDetect^® ^staining of low-intermediate grade CIN. Note that the red staining neoplasia was conspicuously laminar, a feature facilitating grading even at low magnification. (B) H&E-stained section adjacent to A. (C) CellDetect^® ^staining of high grade CIN. (D) H&E-stained section adjacent to C. (E) CellDetect^® ^staining of high grade CIN seen at higher magnification than C, to illustrate details of cell morphology. (F) H&E-stained section adjacent to E.

Normal cervical squamous epithelium and reactive processes of squamous epithelium (see below) stain green/blue (Figure 1). In contrast, morphologically recognizable neoplastic cells in CellDetect^®^-stained preparations always exhibited red/magenta tinged cytoplasm (Figure 2A, C, E). Thus, it was possible to recognize neoplasia, even at low magnification, based exclusively on tinctorial status of the epithelium (Figure 2A, C). Similarly, staining with CellDetect^® ^highlighted the patchy distribution of CIN along the epithelium, a feature we exploited for another aspect of the analysis, considered below. The tinctorial aspects of the histology also clearly demarcated the laminar patterning that is so characteristic of intra-epithelial neoplasia (Fig 2A). Importantly, such color-based impressions were readily confirmed at higher magnification using the same preparation. This could be accomplished because the CellDetect^® ^stain preserved critical diagnostic cytological features of neoplasia evident in the red-stained cells (Figure 2E).

### Quantitative diagnostic performance of CellDetect^®^

Three aspects of diagnostic performance were assessed: (1) diagnostic agreement between the pathologists on each case; (2) the agreement between H&E and CellDetect^® ^for each case, for each pathologist. (3) Micro-regional evaluations directly comparing morphological performance (H&E, CellDetect^®^) with tinctorial characteristics of the same cell populations.

#### Inter-observer variation for case diagnosis

The final diagnosis rendered on each case by each pathologist, using both stains together, was different in 2 cases (~3%) cases (Table 1). These discrepancies were not related to stain used, as documented in the next section.

**Table 1:** Comparison of Pathologists' assessment using H&E and CellDetect^®^

	Pathol #1		Pathol #2	
Diagnosis	H&E	CD	H&E	CD
Normal	21/60*	21/60*	22/60	22/60

CIN	22/60	22/60	22/60	22/60

SCC	17/60	17/60	16/60	16/60

#### Within-observer results between stains

In contrast to the foregoing, each pathologist was entirely consistent across stains, so that no discrepancy between H&E and CellDetect^® ^was noted in any case (Table 1), i.e. each pathologist demonstrated a 100% inter-stain concordance. These data served as a validation, enabling a more detailed assessment of the correlations between morphological and tinctorial diagnosis, as explained in the following sections. Despite this final outcome, CellDetect^® ^highlighted inconspicuous neoplasia in three cases that had initially been overlooked, thus prompting a re-examination of the H&E and revision of the diagnosis according to the CellDetect^® ^findings.

#### Morphology vs. tinctorial characteristics in neoplastic micro-regions

To more critically compare the performance of CellDetect and H&E, the inherent variation of epithelial phenotype often seen in cervical biopsies samples was utilized. A series of 130 microscopic regions with homogeneous phenotype, culled from the 60 cases, was examined by a single pathologist. The pathologist first diagnosed the focus as either normal, CIN or SCC based on H&E. Subsequently, the same area was identified morphologically in the CellDetect^® ^slide, and the tinctorially features were recorded. In all cases of CIN or SCC, cells stained pink/red, so that the false negative rate was 0%. In contrast, loci considered morphologically normal in both H&E and CellDetect^®^, stained green/blue in approximately 90% of cases, i.e. a 10% false positive rate was observed. Table 2 shows the breakdown of the morphological status of each micro-region and the accompanying histochemical results. For each micro-region, staining color was recorded for morphologically normal and atypical cells. By definition, normal cells were not found in CIN3 or SCC micro-regions, and atypical cells were not noted in morphologically normal micro-regions.

**Table 2:** Relation of neoplastic morphology to histochemical results

Dx	n	Normal		Atypical	
		
		Green	Red	Green	Red
Normal	47	89%	11%	-	-

CIN 1*	6	100%	0%	0%	100%

CIN 2	17	88%	12%	0%	100%

CIN 3	23	-	-	0%	100%

SCC	37	-	-	0%	100%

**Total**	**130**				

n, number of micro-regions with diagnosis

* CIN1 fields were found in cases diagnosed as CIN2/3

It is important to note, that although Table 2 lists micro-regions of CIN1, these were incidentally present in cases actually diagnosed as CIN2/3. CIN1 was recognized based on accepted cytological criteria including nuclear atypia and koilocytosis. Since CIN1 is characterized by atypical squamous cells confined to the lower one third of the epithelium, the red staining from CellDetect^® ^was limited to the same extent.

##### Morphology vs. Tinctorial characteristics in reactive micro-regions

To examine the specificity of the histochemical staining reaction to neoplasia, micro-regions were chosen with morphological characteristics on H&E of either inflammation or squamous metaplasia. Table 3 summarizes these observations, and provides the data on normal cells (derived from Table 2), for comparison. It can be seen that the majority of micro-regions with any of these diagnoses was most likely to be green stained. The small percentage of red-stained cells among cells exhibiting squamous metaplasia is similar to their incidence in morphologically normal squamous epithelium.

**Table 3:** Relation of reactive morphology to histochemical results

Dx	n	Green	Red
Normal	47	89%	11%

Inflammation	5	100%	0%

Metaplasia	7	86%	14%

**Total**	**59**		

## Discussion

This work represents the results of a first order clinical assessment of the new dual-function CellDetect^® ^method. This technological approach claims to tinctorially discriminate neoplastic cells while preserving morphology [5]. Using these morphological capabilities, in conjunction with H&E, cytologically neoplastic cells uniformly exhibited a neoplastic *histochemical *phenotype with red/pink cytoplasm, i.e. providing a zero false negative rate.

In contrast, some false positive staining occurs. We found that 10-15% of morphologically *normal *squamous cell foci had cells that stained red/pink, instead of the expected green/blue. On one hand, this raises the exciting possibility that the CellDetect^® ^method is detecting pre-neoplastic changes, heralded at this early stage by some, yet to be defined, metabolic alteration (see below). On the other hand, it is still possible that an artifactual, red staining reaction may occur in some normal cells. In any event, the low level of this phenomenon should not affect the application of CellDetect^® ^to routine diagnostic pathology because it will merely prompt a re-examination of the morphology in such false positive instances.

Another significant implication of this phenomenon is that the same frequency of red-staining micro-regions was seen in metaplastic and inflammatory squamous settings. Although relatively few of such cases were available, the congruence of the findings with the larger sample of normal loci supports the likelihood that these conditions will usually stain differentially as normal tissue, suggesting a high level of specificity of the technology for neoplasia. Naturally, all of these novel findings need to be substantiated in a larger, blinded investigation, which will also include the evaluation of additional benign conditions such as atrophy and immature squamous metaplasia.

From a qualitative perspective, it is worth underscoring that CellDetect^®^, proved particularly useful because the expression of cervical intra-epithelial neoplasia (CIN) is often spotty, and all foci may not be uniform in grade. Indeed, small foci of CIN were occasionally overlooked by our two experienced pathologists using the standard hematoxylin-eosin stained preparations. CellDetect^® ^staining detected neoplasia in these cases. This prompted re-examination of the H&E and revision of the diagnosis, thus confirming the heightened detection sensitivity of the CellDetect^® ^technology. We suspect that the basis for this superior sensitivity is entirely visual. The process of CIN is expressed as a laminar cellular change in the epithelium. CellDetect^® ^staining highlights these changes by forming an intra-epithelial tangential reddish band. The latter is easily seen even at low magnification.

The finding of a ~3% variation in case diagnosis, between the two experienced pathologists, is attributed to well known factors. These include differences in diagnostic criteria for a given grade of neoplasia, or threshold for the diagnosis of neoplasia over normal [7-11]. What is critical to note here is that these differences of opinion were not influenced by the stain used; H&E and CellDetect^® ^had the same outcomes for each observer.

In short, the findings indicate the likelihood that CellDetect^® ^preparations will continue to prove as useful as H&E, and perhaps ultimately, superior because CellDetect^® ^provides both morphological and tinctorial information that are diagnostically relevant.

Given the success of the CellDetect^® ^technology, it is of interest to consider how its performance compares to other cellular markers for cervical neoplasia. For instance, one alternative approach is based on immunocytochemical detection of cellular proteins, such as p53, p16 or HPV viral antigens [12-15 ]. Reports using these markers show that although the marker may be expressed in all grades of cervical neoplasia, in some cases, a particular grade may exhibit no demonstrable expression. That is to say, cells and epithelia satisfying morphological criteria for neoplasia fail to demonstrate the target antigen. In contrast, squamous cells with neoplastic cytology always stained appropriately using CellDetect^®^. An additional complication has been reported regarding the popular p16 (INK4a) in that this marker protein may be demonstrable even in cytologically normal cells [14]. A similar occurrence was found in CellDetect^® ^preparations, but its low incidence should not impact on routine diagnostics, as discussed earlier.

Due to the dramatic, bi-polar histochemical cytoplasmic reaction produced by CellDetect^® ^- red or green - the question of possible mechanism arises. These observations prompt the speculation that the staining phenomenon reflects fundamental shifts in cell structure and function that underlie the transformation from normal/reactive states to neoplasia. For instance, most malignancies characteristically exhibit alternations in metabolic state [16-18], level of differentiation and rates of proliferation [19]. Our previous work suggested a relationship between staining outcome and differentiation. In that study [5], forced differentiation of malignant cell lines uniformly converted the red/magenta cytoplasmic staining to green, as we would anticipate. On the other hand, proliferation of phenotypically benign cells does not alter staining. The latter is exemplified by the proliferative basal cell layer, which always exhibits a non-neoplastic histochemical phenotype, viz. green cytoplasm. Although these observations are provocative, a more direct examination of these issues will be required before any mechanistic option can be accepted.

## Conclusions

In conclusion, the findings in this non-blinded study substantially support the claim that CellDetect^® ^can tinctorially discriminate neoplastic cells while preserving morphology [5], at least for cervical neoplasia. Despite interpretative limitations imposed by a non-blinded experimental design, the outcomes using different strategies for comparison of morphology and tinctorial qualities, were entirely consistent throughout the study Clearly, these initial observations are promising, but they need to be cautiously assessed, and bolstered by a more extensive, prospective evaluation.

## Abbreviations

CIN: Cervical intraepithelial neoplasia; SCC: Squamous cell carcinoma; H&E: haematoxylin and eosin stain; HPV: human papillomavirus;

## Competing interests

Among the authors, Pavel Idelevich, Adi Elkeles, Dov Terkieltaub and Ilia Rivkin are employees of Zetiq; Elimelech Okon, Don Kristt, Ami Fishman are paid consultants.

## Authors' contributions

PI: Developed CellDetect^® ^technology, substantially contributed to conception, design, and analysis of data. Approved published version.

AE: Managed clinical trial and data analysis. Approved published version.

EO: Substantially contributed to data analysis. Approved published version.

DK: Contributed to data analysis and writing the manuscript. Approved published version.

DT: Contributed to data management and analysis. Approved published version.

IR: Carried out CellDetect^® ^staining. Approved published version.

IB: Involved in clinical design and management. Approved published version.

AF: Substantially involved in clinical design, management and analysis. Approved published version.

## Authors' information

Dr. Pavel Idelevich

Dr. Idelevich trained in internal medicine and pathology at Saratov State Medical Institute, and the Soroka Medical Center in Beersheva, where he gained clinical experience training as a medical doctor in GI and urology units. He served as a researcher in the Immunology Department at the Weizmann Institute of Science. An experienced pathologist, Dr. Idelevich has played a central role in the development of Zetiq`s staining technology.

Dr. Adi Elkeles

Dr. Elkeles holds a PhD from Tel Aviv University, an MBA from the College of Management, and completed a three-year post-doctoral fellowship in Molecular Cell Biology at the Weizmann Institute of Science. An alumni of the Paul Merage life science executive leadership program at UC Irvine California, U.S.A, Dr. Elkeles is an experienced executive in the biotech field, specializing in biopharmaceutical development. Dr. Elkeles has significant research and development experience in various fields of biology including cancer, immunology and infectious diseases. Prior to joining Zetiq, Dr. Elkeles served as Drug Development Manager for BiolineRx and as manager at XTL Biopharmaceuticals.

Prof. Elimelech Okon

Prof. Okon is a Clinical professor of Pathology Emeritus at the Sackler School of Medicine, Tel Aviv University. He served as the Chairman of the department of Pathology at Rabin Medical Center between 1996 and 2008. Since 2009, he is the Medical Director at LEM laboratories, Israel.

Prof. Don Kristt

Dr. Kristt is head of Molecular Pathology Unit at the Rabin Medical Center and an adjunct professor at Bar Ilan University. As a physician experienced in both clinical and experimental pathology, he has made important contributions in the fields of histochemistry, electron microscopy, molecular pathology and transplantation medicine. In the past he has been a Professor at Johns Hopkins MC, Stanford MC and the University of Maryland.

Mr. Dov Terkieltaub

A graduate of Bar Ilan University, Dov has extensive experience in product development. A veteran of 18 years in the Biotech field, Dov has managed the development of many products, assays and processes. Experienced in project management, he has brought a number of products from bench to bedside. Prior to joining Zetiq, Dov served at Interpharm ltd (a Serono company) in QC and development, and held managerial positions at XTL Biopharmaceuticals and Intelect Neurosciences.

Mr. Ilia Rivkin

A PhD student at Tel Aviv University, Ilia holds an M. Sc. in Biochemistry from Tel Aviv University and B.Sc. in Biotechnology from Braude Academic College of Engineering. Mr. Rivkin has research and practical experience in fields of chemistry and biotechnology including cell-based assay development, HTS robotic automatization and establishing of drug-carrier systems for cancer treatment.

Dr. Ilan Bruchim

A graduate from Faculty of Medicine, Technion, Haifa, Dr. Bruchim completed his residency in Obstetrics & Gynecology at Meir Medical Center, Kfar-Saba, Israel and a fellowship in Gynecologic Oncology at McGill University, Montreal, Canada. He is currently a faculty member of Gynecologic Oncology Unit, Department of Obstetrics & Gynecology, Meir Medical Center, Kfar-Saba, affiliated with the Sackler School of Medicine, Tel Aviv University.

Prof. Ami Fishman

Graduated from the Faculty of Medicine, Tel-Aviv University. Prof. Fishman completed his residency in Obstetrics & Gynecology at Rambam Medical Center, Haifa, Israel and fellowship in Gynecologic Oncology at Baylor College of Medicine, Houston, TX. In his current position, Prof. Fishman is the Chairman of the Department of Obstetrics & Gynecology, Meir Medical Center, Kfar-Saba, affiliated with the Sackler School of Medicine, Tel-Aviv University, Israel
